# Serum Levels of MicroRNA-371a-3p (M371 Test) as a New Biomarker of Testicular Germ Cell Tumors: Results of a Prospective Multicentric Study

**DOI:** 10.1200/JCO.18.01480

**Published:** 2019-03-15

**Authors:** Klaus-Peter Dieckmann, Arlo Radtke, Lajos Geczi, Cord Matthies, Petra Anheuser, Ulrike Eckardt, Jörg Sommer, Friedemann Zengerling, Emanuela Trenti, Renate Pichler, Hanjo Belz, Stefan Zastrow, Alexander Winter, Sebastian Melchior, Johannes Hammel, Jennifer Kranz, Marius Bolten, Susanne Krege, Björn Haben, Wolfgang Loidl, Christian Guido Ruf, Julia Heinzelbecker, Axel Heidenreich, Jann Frederik Cremers, Christoph Oing, Thomas Hermanns, Christian Daniel Fankhauser, Silke Gillessen, Hermann Reichegger, Richard Cathomas, Martin Pichler, Marcus Hentrich, Klaus Eredics, Anja Lorch, Christian Wülfing, Sven Peine, Werner Wosniok, Carsten Bokemeyer, Gazanfer Belge

**Affiliations:** ^1^Asklepios Klinik Altona, Hamburg, Germany; ^2^ Albertinen-Krankenhaus Hamburg, Hamburg, Germany; ^3^ University of Bremen, Bremen, Germany; ^4^ National Institute of Oncology, Budapest, Hungary; ^5^ Bundeswehrkrankenhaus Hamburg, Hamburg, Germany; ^6^ St Elisabeth Krankenhaus, Leipzig, Germany; ^7^ St Franziskus Hospital, Lohne, Germany; ^8^ University of Ulm, Ulm, Germany; ^9^ Krankenhaus Bozen, Bolzano, Italy; ^10^ Medical University Innsbruck, Innsbruck, Austria; ^11^ Zeisigwaldkliniken, Chemnitz, Germany; ^12^ Universitätsklinikum Carl Gustav Carus, Dresden, Germany; ^13^ Carl von Ossietzky University, Oldenburg, Germany; ^14^ Klinikum Bremen-Mitte, Bremen, Germany; ^15^ St Antonius Hospital, Eschweiler, Germany; ^16^ Ameos Klinikum, Geestland, Germany; ^17^ Klinikum Essen-Mitte Huyssenstiftung, Essen, Germany; ^18^ St Marienkrankenhaus, Ahaus, Germany; ^19^ Ordensklinikum Barmherzige Schwestern, Linz, Austria; ^20^ Bundeswehrzentralkrankenhaus, Koblenz, Germany; ^21^ Universitätsklinikum Saarland, Homburg, Germany; ^22^ Universitätsklinikum Cologne, Cologne, Germany; ^23^ Universitätsklinikum Münster, Münster, Germany; ^24^ Universitätsklinikum Eppendorf, Hamburg, Germany; ^25^ Universitätsspital Zürich, Zurich, Switzerland; ^26^ Kantonsspital, St Gallen, Switzerland; ^27^ Kantonsspital Graubünden, Chur, Switzerland; ^28^ Medical University Graz, Graz, Austria; ^29^ Rotkreuzklinikum, Munich, Germany; ^30^ Kaiser Franz Josef Spital, Wien, Austria; ^31^ Urologische Universitätsklinik der Heinrich Heine Universität, Düsseldorf, Germany

## Abstract

**PURPOSE:**

Previous studies suggested that serum levels of microRNA (miR)-371a-3p (so-called M371 test) have a much higher sensitivity and specificity than the classic markers of testicular germ cell tumors (GCTs) and are applicable toward both seminoma and nonseminoma. We sought to confirm the usefulness of this test as a novel biomarker for GCT.

**PATIENTS AND METHODS:**

In a prospective, multicentric study, serum samples of 616 patients with testicular GCTs and 258 male controls were examined for serum levels of miRNA-371a-3p (miR levels) by quantitative polymerase chain reaction. The GCT population encompassed 359 patients with seminoma and 257 with nonseminoma; 371 had clinical stage I disease, 201 had systemic disease, and 46 had relapses. Paired measurements before and after orchiectomy were performed in 424 patients; 118 with systemic disease had serial measurements during treatment. miR levels were compared with those of β-human chorionic gonadotropin, α-fetoprotein, and lactate dehydrogenase.

**RESULTS:**

For the primary diagnosis of GCT, the M371 test showed a sensitivity of 90.1%, a specificity of 94.0%, an area under the curve of 0.966 upon receiver operating characteristic analysis, and a positive predictive value of 97.2%. α-Fetoprotein, β-human chorionic gonadotropin, and lactate dehydrogenase had sensitivities of less than 50% in seminoma and slightly higher sensitivities in nonseminomas. miR levels were significantly associated with clinical stage, primary tumor size, and response to treatment. Relapses had elevated miR levels that subsequently dropped to normal upon remission. Teratoma did not express miR-371a-3p.

**CONCLUSION:**

The M371 test outperforms the classic markers of GCT with both a sensitivity and a specificity greater than 90%. All histologic subgroups, except teratoma, express this marker. The test could be considered for clinical implementation after further validation.

## INTRODUCTION

The serum tumor markers β-human chorionic gonadotropin, α-fetoprotein (AFP), and lactate dehydrogenase (LDH) became essential tools in the clinical management of testicular germ cell tumors (GCTs) in the late 1970s.^1,2^ Current guidelines recommend the use of marker measurements for clinical staging, treatment monitoring, and follow-up of patients with GCTs.^3-6^ One major drawback of the markers, however, is their low overall sensitivity. Only 50% of all GCTs express one of the three markers, and seminomas lack AFP expression entirely.^7,8^ Moreover, LDH expression is also found in several other diseases.^9^

The use of serum levels of microRNAs (miRs) from the miR-371-3 and miR-302/367 clusters as novel GCT biomarkers was first suggested in 2011.^10^ Generally, miRs represent small noncoding RNAs that are involved in the epigenetic regulation of gene expression.^11^ Previous studies suggested a high sensitivity (> 80%) and specificity (> 90%) of miR-371-3 and miR-302/367 for GCTs, with miR-371a-3p proving to be the most sensitive and most specific.^12-15^ The serum levels of this miR seem to be associated with both clinical stage (CS) and tumor bulk, with levels dropping to normal with a half-life of less than 24 hours after the cancer is cured.^16,17^ Of note, seminomas were found to express miR-371a-3p in more than 85% of patients.

The current consensus is that measurements of miR-371a-3p, also called the M371 test, greatly outperform the classic markers, and thus, a clinical implementation of the test seems warranted. However, the available data are based solely on seven independent small- to moderate-sized studies with retrospective and prospective modes of patient accrual as well as divergent miR measurement techniques.^13,14,18-22^ Moreover, the majority of patients examined thus far have been in the early CSs, and only a few had advanced stages. Accordingly, the body of evidence is both limited by and open to bias. Therefore, the aim of the current study was to prospectively evaluate the utility of the M371 test in a large and representative patient population enrolled from a large number of European institutions and to involve various histologies and CSs. In particular, we aimed to evaluate the diagnostic sensitivity and specificity of the test for the primary diagnosis of GCT and to assess its usefulness for monitoring GCT treatment.

## PATIENTS AND METHODS

### Study Design and Participants

**FIG 1.**
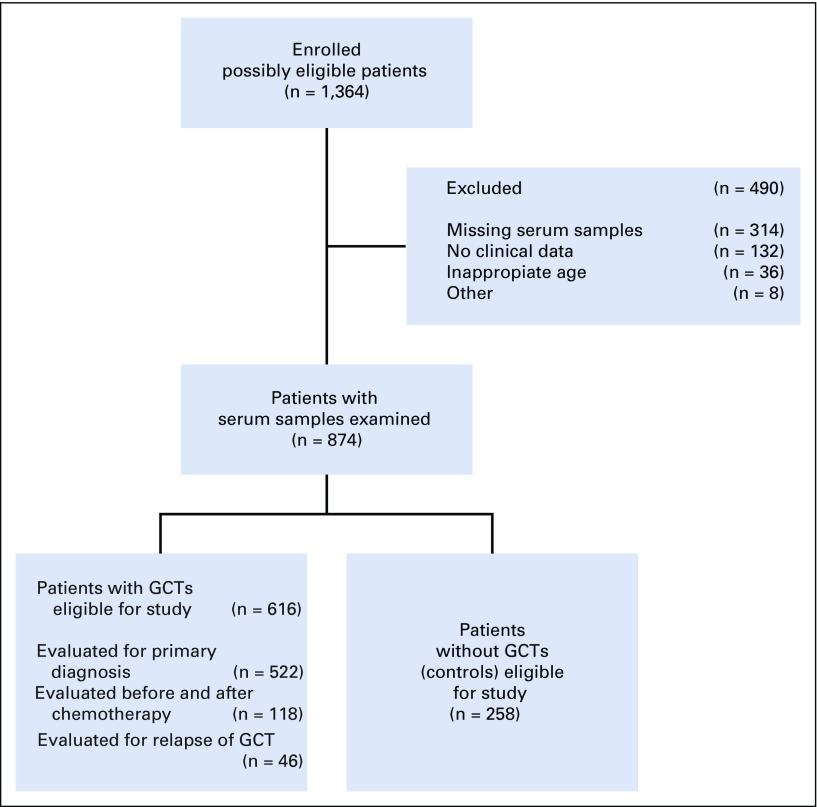
Study profile. The diagram shows the selection process of patient enrollment. GCT, germ cell tumor.

We performed the prospective study at 37 institutions in Germany, Austria, Switzerland, Hungary, and Italy between September 2015 and December 2016 (Appendix Table A1, online only). A total of 1,364 consecutive male patients ages 16 to 69 years were recruited. We excluded 490 patients from the study for various reasons (Fig 1). The final study population consisted of 616 patients with GCT and 258 controls (Table 1). Of the patients with GCT, 522 provided preoperative samples, and 118 with systemic disease underwent repeated sampling over the course of chemotherapy. Controls consisted of 133 males ages 18 to 60 years who presented with nonmalignant testicular disease (NMTD) and 125 healthy male blood donors of the same ages. The rationale for the sample size is detailed in the Appendix (online only). The following patient-related data were registered: date of blood aspiration, patient age, histology, primary tumor size, local pathologic stage (pT), CS according to the Lugano classification, serum levels of classic tumor markers, and treatment received. No information was available with regard to follow-up examinations of the included patients. All patients gave informed consent. The study received ethical approval by Ärztekammer Bremen (#301, 2015).

**TABLE 1.**
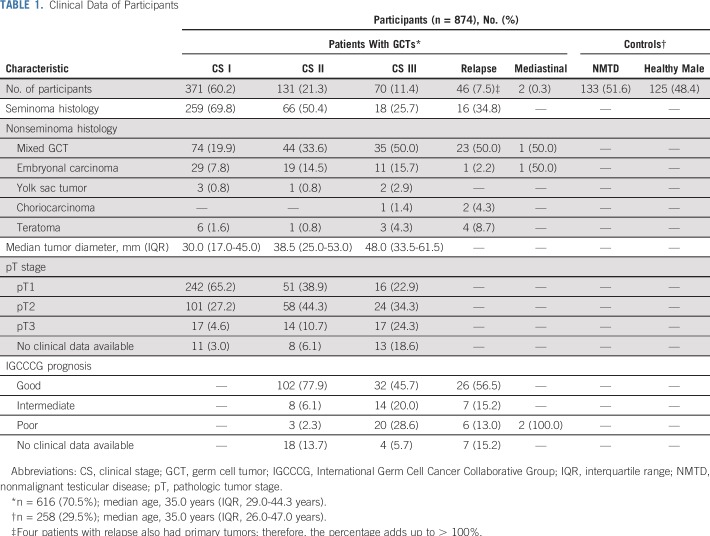
Clinical Data of Participants

### Laboratory Methods

For the measurement of serum miR-371a-3p levels, we used the method described previously.^22^ Briefly, RNA was isolated from cubital vein serum, and then reverse transcription was performed for both miR-371a-3p and the endogenous control miR-30b-5p to cDNA. Quantitative polymerase chain reaction was done after preamplification. Measurement results were documented as relative quantity (RQ) values. Laboratory details are provided in the Appendix.

### Statistical Methods

A two-sided Mann-Whitney *U* test was used to assess differences between two unrelated groups of samples. A Wilcoxon signed rank test was applied for the comparison of repeated measurements in individual patients. Receiver operating characteristic (ROC) analysis was performed with empirical data, and the optimal cutoff value (the highest Youden index) was determined. Sensitivity, specificity, positive predictive value (PPV), negative predictive value (NPV), and positive and negative likelihood ratios were calculated. For the calculation of predictive values, only patients from four large primary care urologic institutions were evaluated (see Appendix for rationale). Kernel density estimation was used as a model of the RQ distribution in an unlimited sample size. The 95% CIs for discriminative measurements derived from density estimation were calculated by bootstrapping, with 2,500 simulations. Differences among categorical data were calculated with an exact χ^2^ test. To test the association between tumor diameter and miR-371a-3p expression, linear regression was used. The association of tumor diameter and sensitivity was tested with logistic regression. RQ values were log-transformed for kernel density estimation and regression analysis, whereby values of 0 were assumed to be equivalent to 0.001. Bonferroni correction was applied to the comparison of miR-371a-3p sensitivity with the classic markers to adjust for multiple testing. All tests were two-sided, and significance was assumed at *P* < .05. Statistical analyses were performed using SPSS version 24 (IBM Corporation, Armonk, NY) or R version 3.2.3 (The R Foundation, Vienna, Austria) software.

## RESULTS

### Evaluation of the M371 Test for Primary Diagnosis of GCT

**FIG 2.**
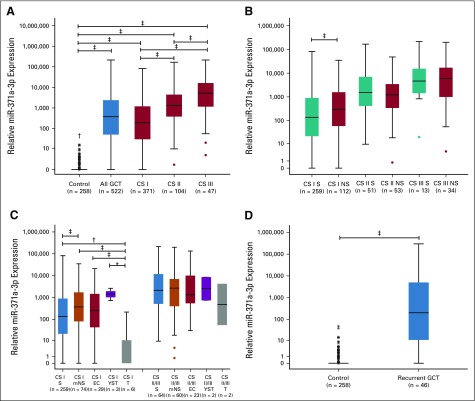
Relative expression of microRNA (miR)-371a-3p in patients with germ cell tumors (GCTs) and controls. (A) Box plots that represent miR-371a-3p expression in controls, patients with GCTs stratified for clinical stages (CSs) I, II, and III. (B) Differential expression of miR-371a-3p in seminoma (S) and nonseminoma (NS) in various CSs. (C) Marker expression in various histologic subtypes for CS I and CS II/III patients. Box plots represent S, mixed NS (mNS), embryonal carcinoma (EC), yolk sac tumor (YST), and teratoma (T). (D) Expression of miR-371a-3p in controls and patients with GCT recurrence. The *y*-axis is arranged in a logarithmic scale in all panels. (*) *P* < .05; (†) *P* < .01; (‡) *P* < .001.

The median expression of miR-371a-3p was significantly higher in the entire GCT group and in all the CS subgroups compared with the controls. Patients with CS greater than I had a higher serum level than those with CS I (all *P* < .001; Fig 2A). Seminoma was found to have significantly lower miR-371a-3p values than nonseminoma. However, this difference was only detectable in CS I patients (Fig 2B). Teratoma had the lowest expression values of all subtypes (Fig 2C). Among controls, healthy blood donors and patients with NMTD did not have significantly different median RQ values (*P* = .4).

**FIG 3.**
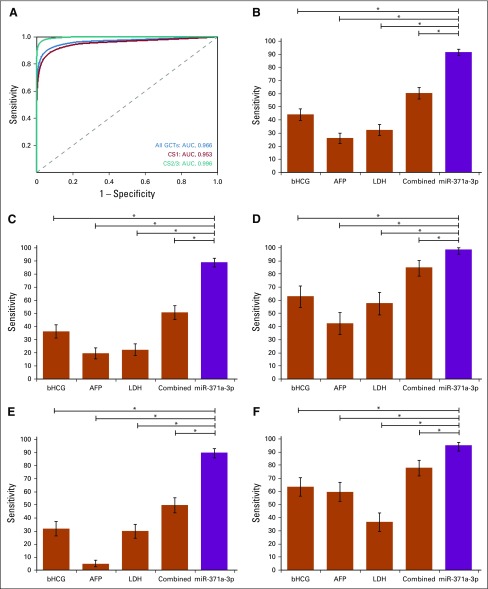
Discriminative ability of microRNA (miR)-371a-3p. (A) Receiver operating characteristic curves that discriminate controls (n = 258) from all patients with germ cell tumors (GCTs; n = 522; area under the curve [AUC], 0.966), clinical stage (CS) I only (n = 371; AUC, 0.953), or CS II/III only (n = 151; AUC, 0.996). (B) Sensitivity of miR-371a-3p in all GCTs (n = 522) compared with the classic GCT markers β-human chorionic gonadotropin (bHCG), α-fetoprotein (AFP), and lactate dehydrogenase (LDH) and all three classic markers combined. (C) Same comparison for CS I GCT only (n = 371). (D) Same comparison for CS II/III GCT only (n = 151). (E) Same comparison for seminoma only (n = 323). (F) Same comparison for nonseminoma only (n = 199). Error bars represent the 95% CI. (*) *P* < .001.

An ROC analysis that was based on preoperative samples of the patients with GCT and controls revealed an area under the curve (AUC) of 0.97 and an optimal cutoff at an RQ of 5 (highest Youden index). On the basis of this cutoff, patients with GCT could be discriminated from controls with a diagnostic sensitivity of 91.8% and a specificity of 96.1%. After using kernel density estimation to model the distribution of RQ values, the AUC was 0.966, whereas the sensitivity was 90.1% and the specificity 94.0% (Fig 3A). PPV was 97.2%, and NPV was 82.7%. Table 2 lists a synopsis of all discriminative parameters for the entire group of GCTs and the subgroups of seminoma and nonseminoma.

**TABLE 2.**
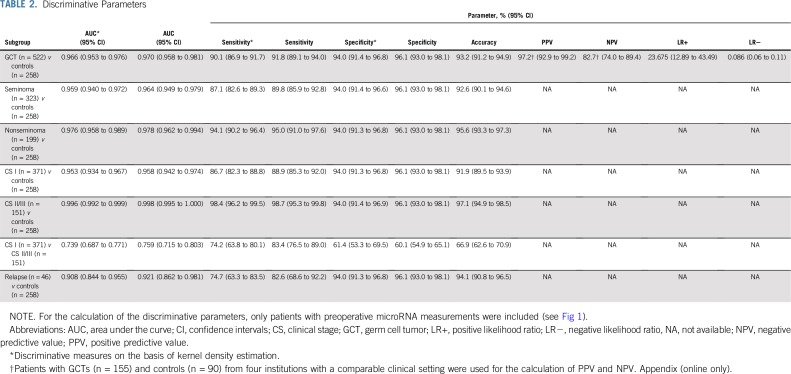
Discriminative Parameters

**FIG A1.**
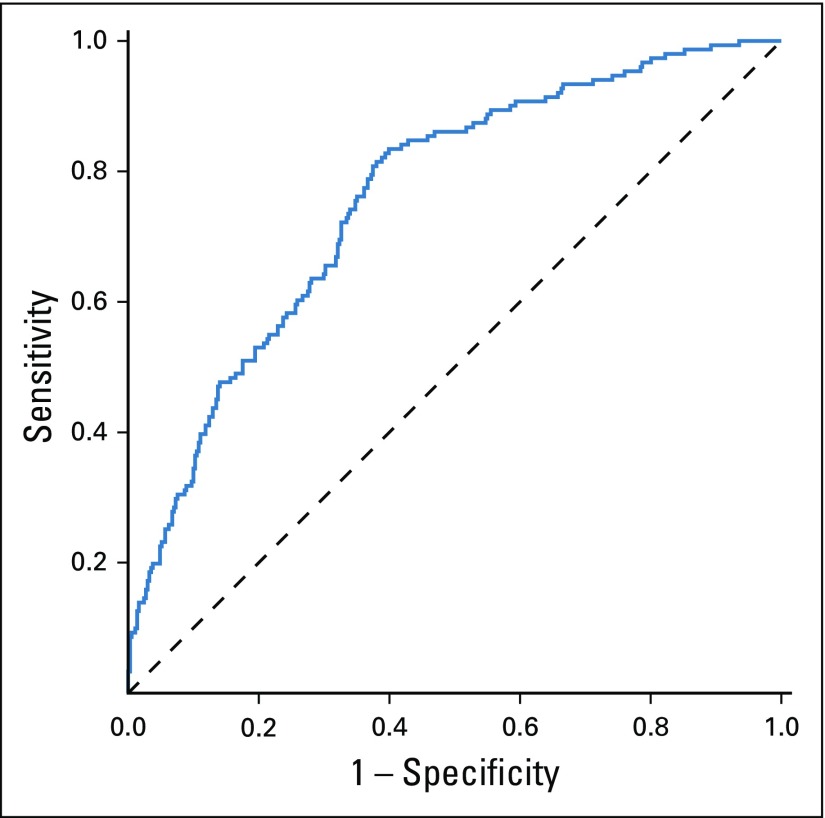
Receiver operating characteristic curve that discriminates clinical stage I patients (n = 371) from patients with systemic disease (n = 151) at an area under the curve of 0.76. A relative quantity cutoff value of 308 (highest Youden index) resulted in a sensitivity of 83.4% and a specificity of 60.1%.

The M371 test discriminated patients with localized GCT (CS I) from those with systemic disease, with an AUC of 0.76 (Appendix Fig A1, online only) and a diagnostic sensitivity and specificity of 83.4% and 60.1%, respectively. Comparison of the sensitivities of the M371 test (empirical data) with the classic GCT markers (Fig 3B) revealed the sensitivity of the new test to be significantly higher than each of the classic markers and even the combination of all three. The superior sensitivity of the M371 test also was found in subgroup analyses of CSs and the two histologic subgroups (Figs 3C to F), with the greatest superiority documented in CS I (Fig 3C) and in seminoma (Fig 3E).

**FIG 4.**
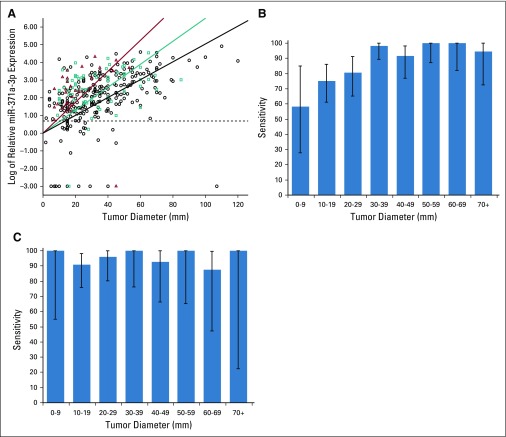
Dependency of microRNA (miR)-371a-3p expression and sensitivity on tumor diameter. (A) Scatterplot that represents the relationship between tumor diameter and the miR-371a-3p expression of clinical stage (CS) I seminoma (circles; n = 259), CS I mixed nonseminoma (squares; n = 74), and CS I embryonal carcinomas (triangles; n = 29). Regression lines are depicted for all three groups in the corresponding color. The dashed line represents the cutoff value. (B) Sensitivity in various categories of tumor diameter of CS I seminoma. (C) Sensitivity in various categories of tumor diameter of CS I nonseminoma. Error bars indicate 95% CI.

**FIG A2.**
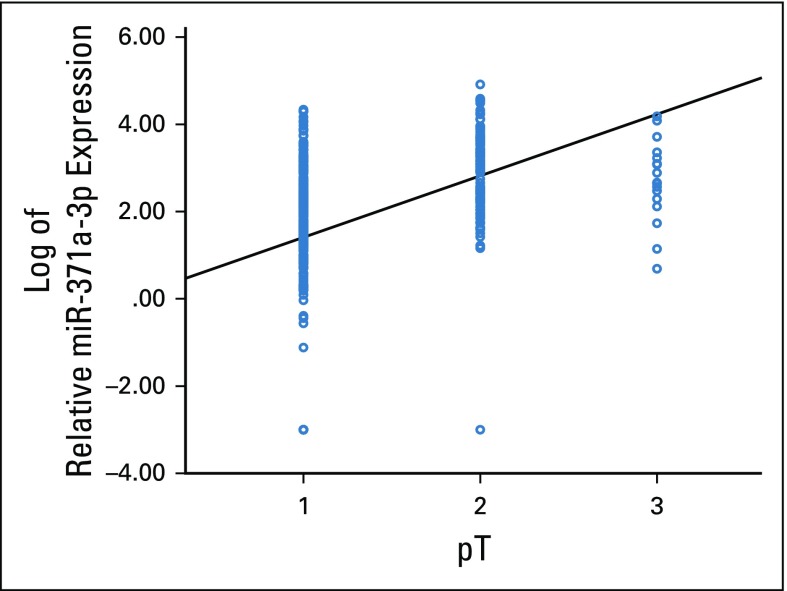
Linear regression analysis revealed an association of microRNA (miR)-371a-3p expression with pathologic tumor (pT) stage (*R*^2^ = 0.664; *P* < .001).

We found a significant regression of tumor diameters with log-transformed miR-371a-3p serum levels (*R*^2^ = 0.653; *P* < .001) in CS I patients. Subgroup analyses also revealed this regression in seminoma, mixed nonseminoma, and embryonal carcinoma (*R*^2^ = 0.686, 0.745, and 0.619, respectively; each *P* < .001; Fig 4A), with much higher slopes of the regression curves in mixed nonseminoma and embryonal carcinoma than in seminoma (Fig 4A). Accordingly, the sensitivity of miR-371a-3p for detecting seminoma was significantly lower in the two lowest tumor size categories (≤ 9 mm and 10 to 19 mm) than in the larger categories (*P* < .001; Fig 4B). In nonseminoma, no divergent sensitivities were found among the various size categories (*P* = .8; Fig 4C). Localized tumors (pT1) had significantly lower median miR levels than advanced local stages (> pT1) among CS I patients (*R*^2^ = 0.664; *P* < .001; Appendix Fig A2, online only).

### miR-371a-3p Levels in Treatment Monitoring

**FIG 5.**
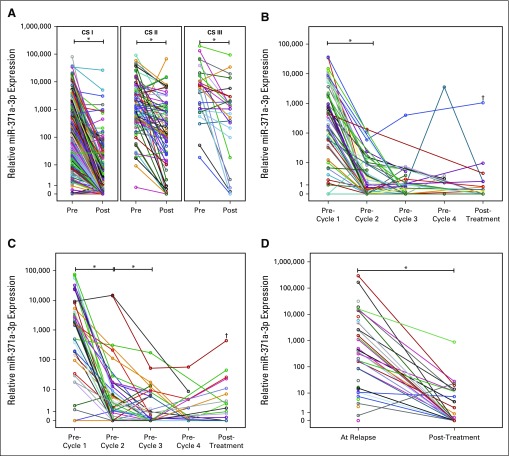
Post-treatment decrease of microRNA (miR)-371a-3p. (A) Decrease of miR-371a-3p expression after surgical removal of the primary tumor in clinical stage (CS) I (n = 316), CS II (n = 80), and CS III (n = 28) patients. (B) miR-371a-3p serum levels over the course of chemotherapy in CS II patients (n = 70). (C) miR-371a-3p serum levels over the course of chemotherapy in CS III patients (n = 46). (D) Decrease of miR-371a-3p expression in recurrent germ cell tumors (GCTs) after treatment (n = 29). Lines in panels B and C are interpolated through missing values. The *y*-axis in all panels is arranged on a logarithmic scale. (*) *P* < .001. (†) Patient with known disease progression and subsequent death.

**FIG A3.**
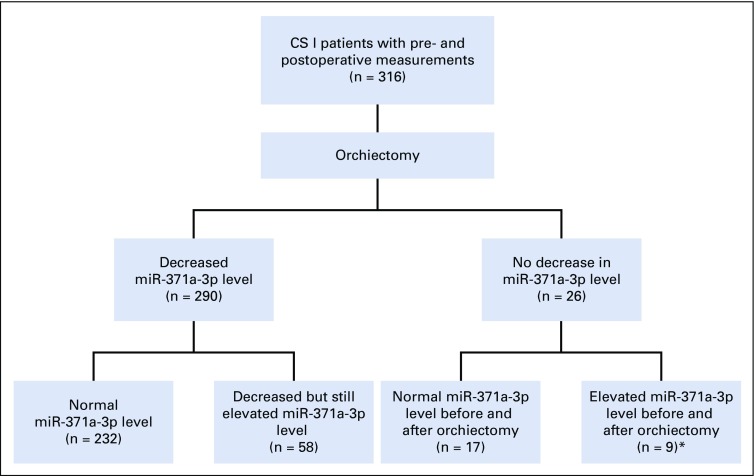
Diagram showing results of paired measurements of microRNA (miR)-371-a-3p levels (before and after orchiectomy) in clinical stage (CS) I patients. (*) Two of the nine patients had elevated levels of classic markers before and after surgery.

Paired measurements of miR-371a-3p in 424 patients before and after orchiectomy revealed a significant drop in miR expression levels in both local and systemic disease (*P* < .001 for each category; Fig 5A). Of the CS I patients, 91.77% had decreased levels after surgery as opposed to 82.41% of patients with metastases (*P* = .008 for proportions of decreasing levels). Appendix Figure A3 (online only) shows additional details.

**FIG A4.**
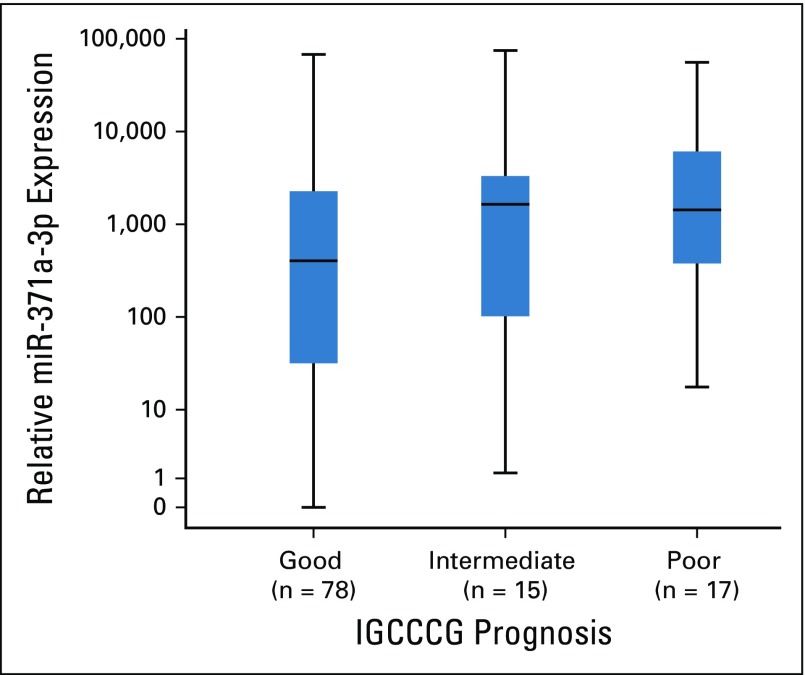
Box plots of microRNA (miR)-371a-3p expression in patients with systemic disease before chemotherapy. Patients with International Germ Cell Cancer Collaborative Group (IGCCCG) poor prognosis have significantly higher levels than patients with good prognosis (*P* = .04).

Serial measurements of miR-371a-3p levels during chemotherapy in 118 patients with systemic disease revealed a significant decrease after the first cycle of therapy. In the 70 patients with CS IIa,b disease, subsequent cycles did not cause a further significant decrease in miR levels (Fig 5B). In CS III patients (n = 46), we observed another significant decrease in miR levels after the second cycle of chemotherapy, with no further significant changes with additional courses (Fig 5C). In two patients with mediastinal GCTs, miR levels dropped upon treatment in the same manner as in CS III patients (data not shown). Two patients had a fatal outcome and achieved strikingly elevated miR levels upon progression (Figs 5B and C as denoted with a †). Patients in the International Germ Cell Cancer Collaborative Group good prognosis category had significantly lower miR-371a-3p median values than those in the poor prognosis category (*P* = .04; Appendix Fig A4, online only).

### miR-371a-3p Levels in Patients With Relapsed Disease

Patients with relapsed disease (n = 46) had a significantly higher median serum miR level than controls (*P* < .001; Fig 2D). Elevated levels were found in 38 patients, which corresponded to a sensitivity of 82.6%, a specificity of 96.1%, and an AUC of 0.921 for relapse detection. Serial measurements during treatment revealed significant decreases in miR levels in 28 of 29 patients (Fig 5D).

## DISCUSSION

This study provides a considerable body of evidence that supports the usefulness of miR-371a-3p serum levels as a new biomarker of GCTs. Five features of the M371 test are noteworthy: The test has a 90.1% sensitivity and a 94.0% specificity for establishing the primary diagnosis of GCT; it is relevant for the two main histologic subgroups of GCT; miR serum levels correlate with primary tumor size, local stage, and CSs; miR levels mirror treatment-related disease changes; and miR levels are elevated in recurrences.

According to current guidelines, the classic tumor markers are not adequately effective for assessing primary diagnosis of GCT because of their low sensitivity and specificity.^4-6^ Currently, clinical and ultrasound examination followed by surgical exploration represent the mainstays of diagnosing GCT. Scrotal magnetic resonance imaging is a second-line diagnostic tool; surveillance can be used in incidentally detected small masses.^23,24^ The high discriminative power of the test is evidenced by an AUC of 0.966 in the ROC analysis, the overall accuracy of 93.2%, and particularly the very high positive likelihood ratio of 23.675 (Table 2). Accordingly, in cases that remain indeterminate despite evaluation with all guideline-recommended examinations, the M371 test may add useful information for clinical decision making before surgery.^16,25^ One foreseeable weakness of the test could be in the detection of pure seminomas with sizes of less than 1 cm because only 59% of these express the marker (Fig 4B).

Serum levels of miR-371a-3p significantly correlate with CS of GCTs (Fig 2A) and with the size of the primary tumor (Fig 4). The most probable explanation for this finding would be a close correlation between miR levels and the amount of tumor (ie, number of cells) present. In fact, this feature is one of the six prerequisites an ideal tumor marker is supposed to have.^2,26^ Accordingly, the M371 test can discriminate locally confined from metastasized disease with a sensitivity of 83.4% but with a specificity of only 60.1%. Computed tomography, the mainstay of clinical staging, has an overall accuracy of 82% with a sensitivity of 59% in detecting retroperitoneal metastases.^5,6,27,28^ Therefore, even in conjunction with the analysis of classic markers, some cases remain unresolved. The M371 test might aid in assessing the correct CS.

In contrast to the classic markers, both of the two main histologic subgroups expressed miR-371a-3p with frequencies of approximately 90%, with the exception of teratoma. In line with previous serum-^14,22,29^ and tissue-based studies,^30,31^ we found CS I patients with pure teratoma to have very low to zero detectable miR-371a-3p expression. Of note, patients with metastasized teratoma displayed elevated miR expression (Fig 2C). Admixtures of other GCT components might have caused the elevation in metastatic teratoma. Nonseminomas have a significantly higher median serum level than seminomas in locally confined disease, and this differential expression has been reported previously but not unanimously.^13,14^ On the basis of the evidence that suggests that the miR-371-3 cluster is expressed primarily by undifferentiated stem cells,^32,33^ it has been hypothesized that the close biologic association between embryonal carcinoma and undifferentiated stem cells results in higher expression of miR-371a-3p in nonseminoma than in the more differentiated seminoma and even less in the well-differentiated teratoma.^22^ Contrasting results were reported from a tissue-based examination wherein seminoma was found to have significantly higher expression of miR-371a-3p than nonseminoma.^30^ However, these findings must be assessed with caution because tissue miR levels do not correlate with serum levels.^18^

An important drawback of the markers AFP, β-human chorionic gonadotropin, and LDH is their overall low sensitivity and low specificity, particularly in the lower CSs.^34,35^ The median miR-371a-3p expression rates are likewise lower in early stages than in advanced disease; however, these differences are small and range between sensitivities of 86.7% and 98.4%. Hence, the M371 test exceptionally outperforms the classic markers at all CSs (Fig 3). Clinically, this feature is most relevant toward the management of seminomas, where more useful tools for the monitoring of patients under surveillance are needed.^36^

The hypothesis that serum miR-371a-3p levels correlate with tumor bulk is supported by our observation that serum levels decreased in response to therapy (Fig 5). After orchiectomy, 91.8% of all CS I patients displayed decreased serum levels, with the majority dropping to within the normal range. The reason why a small proportion of patients had inadequate decreases of miR levels after orchiectomy remains unresolved because there were no follow-up data available in this study. Occult metastatic disease is the most probable hypothesis to explain this finding.

In systemic disease, orchiectomy results in a decrease in miR levels in many patients, although in most, the decrease does not reach the normal range. Marker production by metastatic tissue is the most likely explanation for this observation. Of note, a few CS II patients dropped to normal miR levels postoperatively (Fig 5A). Although the interpretation of this finding is hampered by the lack of follow-up information, a tempting hypothesis would be clinical overstaging in these patients, which would be in accord with surgical studies that documented staging error in approximately 20% of CS II patients.^37^ Serial miR measurements in patients with systemic disease revealed significant decreases upon chemotherapy. Of note, the majority of CS II patients dropped to normal levels after only one cycle, with insignificant further decreases after additional courses (Fig 5B). Because miR levels correlate with tumor bulk, this finding could possibly allow for the hypothesis that the cumulative chemotherapy dosage required for low-volume disease deserves reconsideration.

In CS III patients, there is also a highly significant decrease in miR levels after the first cycle of chemotherapy, with the second cycle producing further significant decreases. At treatment completion, the majority of patients with metastases have normal miR levels. Treatment failure was documented clinically in a few patients, and was accompanied by rising miR levels. The two patients with a lethal outcome displayed increasing miR levels with disease progression and reached the highest values of all patients.

Recently, two independent investigations likewise showed significant decreases of serum M371 levels in patients with metastases who received chemotherapy, which supports the value of this marker for monitoring treatment outcome.^29,38^ Moreover, the Princess Margaret Group demonstrated the value of the marker in identifying viable residual cancer in postchemotherapy residual masses and confirmed the lack of M371 expression in teratoma.^29^

The M371 test has an 82.6% sensitivity at an AUC of 0.921 to detect relapse. One reason why the test may detect recurrences at a somewhat lower sensitivity than primary GCT might be because miR-negative subtypes like teratoma and somatic-type malignancy occur with higher frequency upon recurrence.^27,39,40^ Nevertheless, the M371 test may have the potential to detect recurrences at an early stage in a substantial number of patients as has been recently documented.^16,19,25,41^ MiR levels likewise decreased upon treatment of relapse as they did in metastasized primary disease.

Limitations of the current study stem from the lack of clinical information on patient follow-up because several individual sequences of miR levels could not be sufficiently charted. In addition, histologic work-ups of orchiectomy specimens were performed by local pathologists without central pathology review, which involve minor uncertainties with regard to the differential assessment of nonseminoma. Among the serial measurements of patients with metastases, not all the patients had undergone measurements after each chemotherapy cycle. Accordingly, some missing measurement points may have reduced statistical power. All serum samples were deep frozen for several months until measurement; thus, some sample deterioration may have occurred in the meantime.^42^ Strengths of the study involve the multicentric, multinational design, which resulted in a minimal selection bias. In addition, laboratory measurement techniques were uniformly used for all samples analyzed.

In conclusion, the current study strongly confirms previous data with regard to the usefulness of the M371 test as a new serum biomarker of GCT that is informative in both seminoma and nonseminoma. Because of its high sensitivity and specificity, this test involves the potential of simplifying clinical pathways of the management of GCT, although further validation in an independent cohort is needed.

## Figures and Tables

**TABLE A1.**
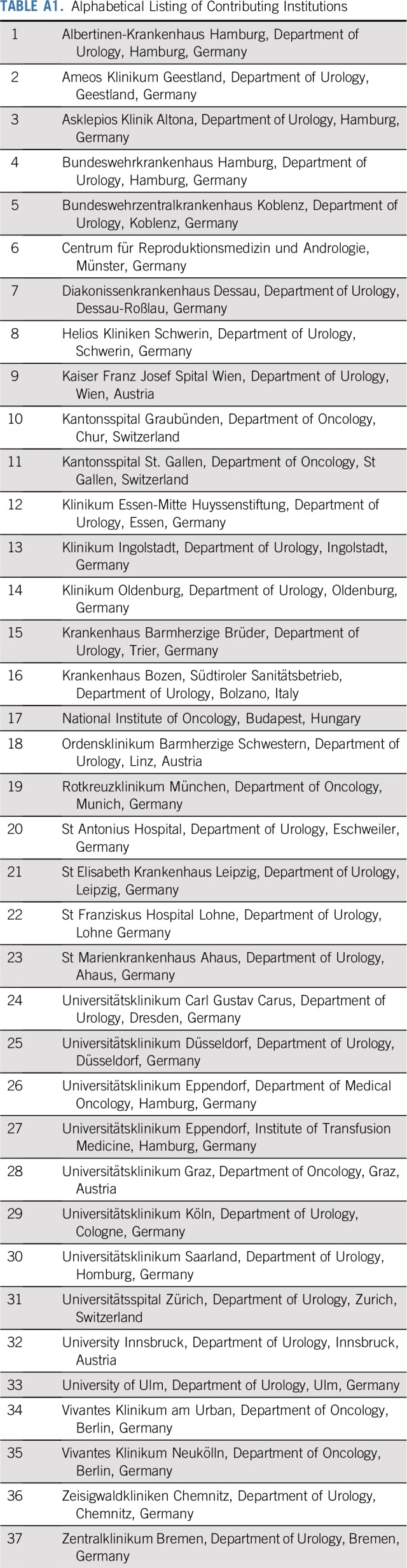
Alphabetical Listing of Contributing Institutions

## Appendix

### Methods

#### Rationale for sample size.

In this study, we aimed to conduct several subanalyses to confirm the usefulness of the M371 test as a germ cell tumor (GCT) serum biomarker. The most important analysis was the comparison of the sensitivity of M371 with that of the classic markers in various histologic subgroups. For this purpose, we used the data available from our previous study on the M371test^22^ and determined the effect size *w* for several comparisons. The smallest effect size was found when all classic markers (combined) were compared with M371 in the subgroup of nonseminoma (*w* = 0.37). To achieve this effect size with a power of at least 0.95 and an α-level of .01 (as a result of Bonferroni correction) in a χ2 test, we would need 90 patients with nonseminoma for analysis of preoperative M371 values. With the assumption of a proportion of nonseminoma of at least 30% among the entire GCT population, we would need to enroll approximately 300 study patients to obtain sufficient numbers with nonseminoma for the preoperative analysis.

Furthermore, we aimed to analyze a meaningful number of patients with pure GCT histologies, particularly pure embryonal carcinoma, to validate the very high M371 expression rates in these subgroups. Because only 10% of GCTs reportedly represent pure embryonal carcinomas (Ulbright TM: Mod Pathol 18:S61-S79, 2005), we decided to increase the sample number to 500 patients to obtain approximately 50 pure embryonal carcinomas for analysis.

The next important analysis was the monitoring of M371 serum values over the course of chemotherapy. Again, the sample size was estimated on the basis of the data from our previous study, and it became evident that we would need at least 90 patients to detect a similar effect size between chemotherapy cycles at a power of 0.95 and an α-level of .05 with a Wilcoxon signed rank test. We therefore aimed to enroll at least 100 patients to provide repeat serum samples taken over the course of chemotherapy.

Finally, we assumed that a significant proportion of the patients originally included would have to be excluded from study in the end because of missing clinical data, which is a common problem in large multicentric clinical investigations. We therefore increased the calculated number of patients to be enrolled by one third to account for dropouts.

#### Exclusion criteria for patients.

The following reasons were applied to exclude participants from the study: age of participant outside the range of 16 to 69 years, substantial clinical data missing (eg, histology, clinical stage), and missing samples at important time points (eg, starting samples in patients treated with chemotherapy).

#### Rationale for selection of institutions for calculation of predictive values.

Principally, to calculate the positive predictive values (PPVs) and negative predictive values (NPVs), a study population that consisted of both patients with and without disease is needed. However, among the participating institutions of the current study, there were marked imbalances with regard to the ratio of patients with tumors and those without tumors (the prevalence). Some of the participating clinics represented tertiary care centers rather than primary care institutions and, therefore, contributed few to no patients without tumors. In addition, there were more patients with tumors than controls who had to be excluded from the study because of missing clinical data.

To have a largely homogeneous study population for calculating PPV and NPV, we decided to use four particular institutions (Albertinen-Krankenhaus Hamburg, Asklepios Klinik Altona, Bundeswehrkrankenhaus Hamburg, and Klinikum Bremen-Mitte), which were selected because they represented primary urologic care units and all contributed substantial numbers of both patients with GCTs and controls. In fact, the majority of study controls with nonmalignant testicular disease (90 of 133) were enrolled by these four institutions. Moreover, only very few of these particular controls had to be excluded. The study population for calculating predictive values thus encompassed 155 patients with GCTs and 90 controls without tumors. The disease prevalence rate was 63.3% (95% CI, 56.9% to 69.3%).

### Laboratory Methods

#### Handling of blood samples.

Whole-blood samples were processed to serum in local hospital laboratories by centrifugation at 2,500× *g*. Serum aliquots were then stored at −80°C. The frozen samples were shipped to the central study laboratory at the University of Bremen where all study material was kept at −80°C until final processing.

#### Measurement of microRNA-371a-3p in serum.

For relative quantification of miRNA (miR)-371a-3p in serum, RNA was isolated from 200 μL cubital vein serum using the miRNeasy Mini Kit (QIAGEN, Hilden, Germany) according to the manufacturer’s instructions. cDNA synthesis was conducted with the TaqMan miRNA Reverse Transcription Kit (Thermo Fisher Scientific, Schwerte, Germany). Stem-loop primers for miR-371a-3p and the endogenous control miR-30b-5p were part of the corresponding TaqMan assays (assay identifiers 002124 and 000602, respectively; Thermo Fisher Scientific). The cDNA was pre-amplified in a standard polymerase chain reaction (PCR) using TaqMan assays in a 1:100 dilution and a hot start master mix (Jena Bioscience, Jena, Germany) with the following temperature profile: 1 minute at 95°C followed by 14 cycles of 15 seconds at 95°C and 4 minutes at 60°C. Thereafter, the miRNA was quantified in 40 cycles of quantitative PCR on a 7500 Fast Real-Time PCR System (Thermo Fisher Scientific) with the aforementioned TaqMan assay and FastStart Universal Probe Master Mix (Roche, Mannheim, Germany). The temperature profile was as follows: 10 minutes at 95°C followed by 40 cycles of 15 seconds at 95°C and 1 minute at 60°C. The relative quantity of miR-371a-3p was calculated according to the ΔΔCT method. Each sample was measured in triplicate. Control measurements without reverse transcription were run for every sample, and no-template controls were run for every experiment.

#### Repeat measurements.

Repeat measurements were performed on six serum samples with five repetitions each. For each sample, the standard deviation of the five measurement values was calculated. All six samples together showed an average standard deviation of quantitation cycle values of 0.542 and 0.45 for miR-371a-3p and the endogenous control miR-30b, respectively. The relative quantity values thus involve an average standard deviation of 19.25%.

#### Measurement of traditional tumor markers.

Serum levels of β-human chorionic gonadotropin, α-fetoprotein, and lactate dehydrogenase were measured according to standard laboratory guidelines in local hospital laboratories.^3^
